# Detection of Banana Mild Mosaic Virus in *Musa* In Vitro Plants: High-Throughput Sequencing Presents Higher Diagnostic Sensitivity Than (IC)-RT-PCR and Identifies a New Betaflexiviridae Species

**DOI:** 10.3390/plants11020226

**Published:** 2022-01-15

**Authors:** Marwa Hanafi, Wei Rong, Lucie Tamisier, Chadi Berhal, Nicolas Roux, Sebastien Massart

**Affiliations:** 1 Integrated and Urban Plant Pathology Laboratory, Gembloux Agro-Bio Tech, University of Liège, 2, Passage des Déportés, 5030 Gembloux, Belgium; wei.rong@uliege.be (W.R.); lucie.tamisier@inrae.fr (L.T.); chadi.berhal@uliege.be (C.B.); 2 Bioversity International, Parc Scientifique Agropolis II, 34397 Montpellier, France; n.roux@cgiar.org

**Keywords:** high-throughput sequencing, (IC)-RT-PCR, diagnostic performance, BanMMV, in vitro plants, meristem culture, RNA extraction

## Abstract

The banana mild mosaic virus (BanMMV) (Betaflexiviridae, Quinvirinae, unassigned species) is a filamentous virus that infects *Musa* spp. and has a very wide geographical distribution. The current BanMMV indexing process for an accession requires the testing of no less than four plants cultivated in a greenhouse for at least 6 months and causes a significant delay for the distribution of the germplasm. We evaluated the sensitivity of different protocols for BanMMV detection from in vitro plants to accelerate the testing process. We first used corm tissues from 137 in vitro plants and obtained a diagnostic sensitivity (DSE) of only 61% when testing four plants per accession. After thermotherapy was carried out to eliminate BanMMV infection, the meristem was recovered and further grown in vitro. The same protocol was evaluated in parallel on the corm tissue surrounding the meristem, as a rapid screening to evaluate virus therapy success, and was compared to the results obtained following the standard protocol. The obtained results showed 28% false negatives when conducting testing from corm tissues, making this protocol unsuitable in routine processes. Furthermore, RT-PCR and high-throughput sequencing (HTS) tests were applied on tissues from the base (*n* = 39) and the leaves (*n* = 36). For RT-PCR, the average DSE per sample reached 65% from either the base or leaves. HTS was applied on 36 samples and yielded 100% diagnostic specificity (DSP) and 100% DSE, whatever the sampled tissue, allowing the identification of a new Betaflexiviridae species infecting *Musa*. These results suggest that a reliable diagnostic of BanMMV from in vitro plants using RT-PCR or HTS technologies might represent an efficient alternative for testing after greenhouse cultivation.

## 1. Introduction

Plant pests are seriously threatening food security worldwide. The damages caused by plant pathogens could reach 40% of food production all over the world [1]. The risk of epidemics is elevating due to climate change and increasing commercial trade [2]. Thus, there is a requirement for virus-tested planting material for guaranteeing the exchange of germplasms and for disease control [3,4]. In this context, the International Plant Protection Convention (IPPC), a plant health treaty signed by over 180 countries, was developed in the 1920s in order to address organisms that are both directly and indirectly harmful to plants. The reliable detection of plant pathogens is therefore a crucial step in the proper management of many diseases and to avoid their geographical extension due to exchanges of planting material [5].

The banana (*Musa* spp.) is one of the most important staple food crops, supplying food to more than 400 million people in more than 136 countries. Its global production is approximately 153 million tons annually [6]. However, pests and diseases greatly contribute to the decline in banana yields. They represent significant threats to banana production and have the potential to devastate entire plantations [1,7]. Among these pests, viruses constitute an important concern to banana and plantain production, as they directly affect the yield and quality. They also remain a serious constraint to the international exchange of *Musa* germplasms [8]. About 20 virus species belonging to five families have been reported to infect bananas and plantains worldwide [8]. Several species of *Banana streak viruses* (BSVs, genus *Badnavirus*, family Caulimoviridae), the *banana bunchy top virus* (BBTV, genus *Babuvirus*, family Nanoviridae), and the *banana bract mosaic virus* (BBrMV, genus *Potyvirus*, family Potyviridae) have caused documented epidemics [8,9], while *Musa* plants can be infected by other viruses such as *banana mild mosaic virus* (BanMMV) and *banana virus X* (BVX), both unassigned members in the family Betaflexiviridae, or *cucumber mosaic virus* (CMV, genus *Cucumovirus*, family Bromoviridae) [8].

Among these banana viruses, BanMMV and BSVs are the most prevalent viruses in germplasm accessions collected from Asia, Africa, Oceania, and America [10]. BanMMV has flexuous filamentous particles of about 580 nm in length, with a coat protein of ca. 26.8 kDa and a single-stranded positive RNA genome [9,11]. The infection often results in symptomless plants of *Musa* spp., and the virus has a worldwide distribution, which can explain its high prevalence [9,10]. The impact of BanMMV seems somewhat mild on banana crop, although mixed infections (mostly with BSV and BBrMV) can lead to severe leaf necrosis [9,11,12]. The virus displays a very high molecular diversity [13]. Its presence in the banana collection must be avoided to prevent the emergence of more virulent strains and to reduce the risk of unpredictable variations in symptoms, infectivity, accumulation, and/or vector transmissibility due to co-infection with other banana viruses [10].

Currently, the indexing protocols require a growth of banana plants for 6 months in a greenhouse to minimize the risk of false-negative results for the diagnostic test. Leaf samples, both limb and midrib from the three youngest leaves, are collected from four individual plants of each accession and then bulked together for testing. PCR and electron microscopy tests are conducted from these samples at two stages: 3 and 6 months of growth in a greenhouse. Thus, reliable testing requires a significant amount of resources and time. Therefore, another detection protocol called “pre-indexing” has been proposed to determine the health status of banana accessions after 3 months in the greenhouse. It consists of preliminary and quick (RT)-PCR tests from only a single plant. A positive plant will be directly processed for virus therapy, while a negative plant will be fully indexed. The pre-indexing protocol saves a significant amount of time, as 68% of the received plants in the international germplasm collection are infected by a virus and they can be sanitized directly without the need of complete indexing [10]. In this context, BanMMV testing directly from in vitro plants, as carried out for other viruses on several crops such as cassava [14], yam [15], potato [16], and sweet potato [17], holds interesting potential to save time and resources. In addition, virus testing by high-throughput sequencing technologies (HTS) represents a promising alternative to PCR-based tests in plant virus diagnostics, with potentially an improved inclusivity for divergent isolates and species and a similar analytical sensitivity [18]. Comparison of HTS performance with RT-PCR holds great interest for improving virus diagnostics [19].

On the other hand, the sanitation process of banana accession includes chemotherapy, thermotherapy, and meristem culture [20]. It is very long and can last from 12 months to several years. One bottleneck is the ability to rapidly and reliably test the presence of the infecting virus BanMMV, as it is currently carried out after in vitro plant recovery and growth and an additional period of at least 3 months in the greenhouse. Evaluating the success of therapy at the earliest stages is particularly important to save resources and quickly eliminate plants that are not sanitized. An interesting possibility is to test the BanMMV presence in the tissue surrounding the meristem that is sampled after thermotherapy. A quick decision of keeping the plant or not could be made at this stage if such a test performs well.

In this study, we evaluated the possibility of using in vitro plantlets to speed up diagnostic tests and alleviate testing and labor costs. Our goals were to: (i) study the reliability of the BanMMV testing (IC-RT-PCR) carried out on corm tissues (material surrounding the meristem) of in vitro plants while preserving the meristem; (ii) compare the performance of a BanMMV diagnostic test (IC-RT-PCR) performed after thermotherapy, either from corm tissue from in vitro plantlets or from the same plants grown 3 months in a greenhouse; (iii) evaluate and compare the performance of a BanMMV diagnostic test (RT-PCR) performed on RNAs extracted from either the base (area regrouping the corm and meristem) or leaves of in vitro plants; and (iv) evaluate the performance of HTS technologies applied on the base or leaves from in vitro plants and to compare it to RT-PCR.

## 2. Materials and Methods

### 2.1. Plant Material and Tissue Culture Conditions

Banana plantlets were multiplied and maintained in sterile conditions on semi-solid growth medium based on Murashige and Skoog (1962) (MS) macronutrients, (MS) micronutrients, and vitamins (Duchefa Biochemie, Haarlem, The Netherlands) with 3% sucrose (Merk KGaA, Darmstadt, Germany) and solidified using 3 g/L Gelrite™ (Duchefa Biochemie, Haarlem, The Netherlands) [20]. Media were supplemented with 10mg/L ascorbic acid (Sigma-Aldrich Co., St. Louis, MO, USA), 1µM of indole-3-acetic acid (IAA) (Duchefa Biochemie, Haarlem, The Netherlands) and 1µM of 6-benzylaminopurine (BAP) (Sigma-Aldrich Co., St. Louis, MO, USA). For all the media, pH was adjusted to 6.12–6.14 using NaOH or HCl prior to autoclaving at 110°C for 20 min.

Plant material originated from the International *Musa* Germplasm Transit Centre (ITC), which is managed by the Alliance of Bioversity International and CIAT, and hosted at KU Leuven in Belgium, where it was conserved under in vitro conditions. In this study, 137 plants from 19 BanMMV-infected accessions were used for in vitro testing (only IC-RT-PCR) of the plant tissue surrounding the meristem that went to meristem therapy. Moreover, the tissue surrounding the meristem was sampled for 41 plants, from 21 accessions, after thermotherapy when the meristem was further grown in vitro. Three banana accessions (9 plants) known to be healthy were also used as negative controls during tests. A detailed list of this plant material is provided in Supplementary File S1. All information about accessions was found in Musa Germplasm Information System (MGIS) database [21].

In addition, seven banana accessions (23 plants) known to be infected with BanMMV were used for testing (RT-PCR and HTS) of either basal or leaf tissues. Four accessions (16 plants) that tested negative for BanMMV infection were also used as negative controls during these tests. Two out of these four accessions were used for HTS. Details are provided in Supplementary File S1.

### 2.2. Sanitation Process

**Figure 1:**
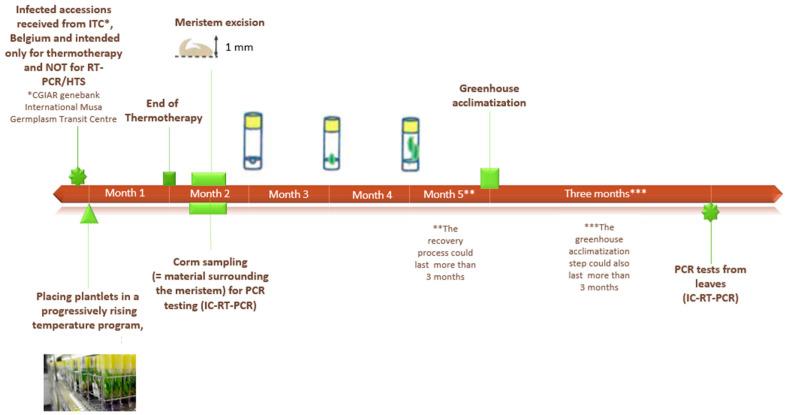
Flowchart of the sanitation process used in this work, describing the different steps involved in the work carried out and the tests performed throughout the experiment.

Twenty-one banana accessions infected with BanMMV (and not available for distribution) and three healthy accessions (ITC0245, ITC0654, and ITC1120) were received from the ITC collection of Bioversity International. Only infected plants were submitted to a cycle of sanitation by thermotherapy with meristem culture (Figure 1).

During the thermotherapy process, infected plants were placed in a temperature-controlled chamber (TCC) with a progressively rising temperature program for one month. This program started at 28 °C, then was increased by 3 °C per day, for 4 days, until reaching 40 °C. Then, the temperature stabilized at 40 °C for 4 weeks.

After the thermotherapy, the apical meristem tip (1 mm diameter) was dissected following the protocol described by Lassois et al. [20] and transferred bi-monthly to a new glass tube containing sterilized fresh growth medium until plantlets had two or three leaves and some roots. This process corresponded to the in vitro stage. Later, plantlets were acclimatized for three months in the greenhouse, at a temperature of 23 ± 2°C and a 16 h/8 h photoperiod. This period of acclimatization was the greenhouse stage (Figure 1).

### 2.3. Sampling of In Vitro Plants

#### 2.3.1. Meristem Sampling

The meristem sampling and in vitro culture were performed in aseptic conditions. Meristem excision was conducted following the protocol described by Lassois et al. [20]. First, successive leaves, overlapping the meristem, were carefully cut with a scalpel through the circular insertion of each one. Then, a binocular microscope was used for a precise excision of the small explant obtained. The apical meristem tip (1 mm diameter) was isolated with a second sterile scalpel and transferred to a new tube with 10 mL of regeneration medium.

#### 2.3.2. Corm Sampling

In vitro tissues surrounding the meristem, the zone henceforth called corm, were sampled at the same time as meristem sampling. In total, 100 mg of the corm was kept for testing while isolating the meristem and preserving it for the in vitro plant regeneration.

#### 2.3.3. The Base Sampling

For some plants, corm tissues and meristem area, the zone henceforth called the base, were sampled and a weight of 100 mg from these tissues was used for total RNA extraction. There was no preservation of the meristem through this sampling. Thus, the plant was killed.

#### 2.3.4. Leaf Sampling

Leaf sampling from in vitro plants was carried out by punching one or two times the three youngest leaves of the plant. A weight of 100 mg from these tissues was used for total RNA extraction. In some cases, the entire leaf was sampled if the weight was under the required weight.

### 2.4. Sampling of Plants in Greenhouse

When the acclimatization period was over, 1/3 of the youngest leaves were sampled from each plant using a disposable sterile scalpel blade. In the laboratory, 8 discs/ sampled leaf were subsampled from both laminar and midrib areas using a water-cleaned, bleached, then water-rinsed 4 mm leaf punch. These samples were directly processed or stored at −80 °C.

### 2.5. RNA Extraction

Total RNA was extracted from in vitro tissues (100 mg in total) of either the base or leaves (separately) using RNeasy Plus Mini Kit (QIAGEN, Hilden, Germany) according to the manufacturer’s instructions. Purified RNA concentration was quantified by spectrophotometry, and quality was evaluated using the Agilent 2100 Bioanalyzer (2100 expert software, version B.02.07.SI532). RNA extracts from each in vitro plant were used for both targeted molecular test (RT-PCR) or high-throughput sequencing.

In addition, two alien external controls were used. They corresponded to plant samples infected at high concentration by a plant virus, called the alien plant virus, which cannot infect banana. They were processed in parallel to the samples from sampling to bioinformatic analysis. The presence of sequences from alien plant viruses in the banana samples can give a useful indication of the cross-contamination level between samples. Therefore, total RNA was also extracted from greenhouse leaf samples (100 mg in total) of one wheat infected by barley yellow dwarf virus (BYDV) as well as a tomato plant infected by Pepino mosaic virus (PepMV). Our two alien viruses were therefore BYDV and PepMV.

### 2.6. Molecular Assays

#### 2.6.1. Targeted Molecular Diagnostic on In Vitro Plants

An IC-RT-PCR test was carried out from crude extracts of corm tissues following the protocol described by De Clerck et al. [10]. Details are included in Supplementary File S2.

For the base and leaf samples, an RT-PCR test was conducted from RNA extracts following the same protocol but without IC.

For IC-RT-PCR and for RT-PCR, a non-template control corresponding to molecular-grade water was used.

#### 2.6.2. Targeted Molecular Diagnostic on Greenhouse Plants

An IC-RT-PCR test was conducted on crude extracts of leaf samples from plants grown in greenhouse for at least three months. The protocol was the same as the one used from in vitro tissues. Details are included in Supplementary File S2.

PCR products were separated by electrophoresis in a 1% agarose gel in 0.5× TBE and stained with GelRed (Biotium). The electrophoresis of PCR products was carried out the same as it was for the IC-RT-PCR test from in vitro plants. Different healthy accessions were used as negative controls for both tests.

### 2.7. Library Preparation and High-Throughput Sequencing

The sequencing libraries were prepared using the Ribo-Zero™ Plant Leaf Kit (Illumina Inc., San Diego, CA, USA) for ribodepletion (ribosomal RNA depletion) followed by the TruSeq Stranded Total RNA Library Prep Kit (Illumina Inc., San Diego, CA, USA) using the standard protocol as previously described [22]. The samples were sequenced on the Illumina Nextseq 500 platform with paired sequencing reads of 2 × 151 nt at the GIGA facilities of Liège University (Liège, Belgium).

### 2.8. Statistical Analyses

The statistical analyses were performed using R software (http://www.r-project.org/, accessed on 1 April 2021). The sensibility was measured for all the possible combinations of 1 to 4 plants using the comb function.

The performance criteria of the diagnostic tests were analysed through the calculation of the diagnostic sensitivity (DSE) and the diagnostic specificity (DSP) of tests, taking into account the status of the accession (healthy or infected) and as follows:

(1) $$diagnostic\;sensitivity\;\left(in\;\%\right)=\left(\frac{True\;positives\;}{True\;positivies+False\;negatives\;}\right)\times 100$$

(2) $$diagnostic\;specificity\;\left(in\;\%\right)=\left(\frac{True\;negatives}{True\;negatives+False\;positives\;}\right)\times 100$$

### 2.9. Bioinformatics Analysis

The obtained sequence reads from HTS were subjected to demultiplexing and removal of Illumina adapter sequences before all reads were paired, quality filtered, and trimmed by BBDuk. In this study, reads shorter than 35 bp and Phred score less than 25 on both ends of reads were trimmed. Later, the trimmed reads were further merged by BBMerge, and “normal” rate was used. All the paired, trimmed, and merged reads were mapped to the custom-built database with all the reference sequences of BanMMV, one reference genome sequence for barley yellow dwarf virus (BYDV, GenBank Accession No. KU170668), and one reference genome sequence for *Pepino Mosaic virus* (PepMV, GenBank Accession No. FJ457096). Two hundred and ten nucleotide sequences of BanMMV were downloaded from NCBI on 12 December 2020. Among them, 54 sequences corresponded to the CP sequences of the virus (40 partial and 14 complete sequences) and 154 corresponded to partial RdRp sequences. A single complete genome of BanMMV (GenBank accession NC_002729) was available. GenBank accessions numbers and names of these sequences are listed in Supplementary File S3.

The mapper “Geneious”, which is a fast mapping method with high sensitivity, was selected. In order to improve the results by aligning reads to each other in addition to the reference sequence, the fine tuning for mapping was set to “Iterate 2 times”. In order to save calculation time and improve mapping efficiency, “20% mismatches” tolerance and customer sensitivity were selected for the sensitivity option for BanMMV, PepMV, and BYDV mapping, respectively. The “map multiple best matches” option was set to “Randomly”, under which the reads will be mapped randomly to one of the best hits presenting equal scoring. A sample was considered positive by HTS if more than 10 reads were mapped to one of the BanMMV downloaded sequences [23].

De novo assembly into contigs was carried out to reconstruct the genome sequence of a potential new viral species, using the SPAdes software embedded in Geneious with default parameters and a k-mer of 55 [22]. The full genome sequence was aligned and used for phylogenetic analyses with the MEGA software package version 7.0. The phylogenetic relationships were inferred using neighbor-joining method embedded in the same version of the MEGA software. The stability of the topology was evaluated using bootstrap (1000 replications) [22]. Accession numbers of virus sequences obtained from GenBank (http://www.ncbi.nlm.nih.gov/, accessed on 1 October 2021) have been integrated in the phylogenetic trees.

### 2.10. Validation of Detection of the New Species

To confirm the sequence of the new species, its RdRp and CP genes were amplified by RT-PCR and sequenced. Firstly, nine primer pairs were designed using Geneious software (v11.0.4). Seven pairs were used to amplify the RdRp of the new sequence, and two pairs were used to amplify the CP of the same sequence. Primers sequences and corresponding PCR programs are detailed in Supplementary File S4. The Tm and dimer formation of the selected primers were also checked using Geneious. Then, Sanger sequencing (Macrogen Europe BV, Amsterdam, The Netherlands) was performed from purified PCR products.

## 3. Results

### 3.1. Evaluation of Diagnostic Sensitivity of BanMMV Detection from Corm Tissues of Banana In Vitro Plants by IC-RT-PCR

#### 3.1.1. Results on In Vitro Plants

According to these results, 52 out of 137 plants tested positive (Table 1). Thus, 38% of overall diagnostic sensitivity has been recorded. The percentage of detection per accession varied from 0 to 100%, depending on the accession. Among the 19 accessions tested, six showed 0% of virus detection, and only one showed 100% of detection. Some results of this testing are shown in Supplementary File S5.

The six BanMMV-infected accessions, for which 0% of virus detection was recorded, had different geographical origins. Details are provided in Supplementary File S1. This downplays the hypothesis that the undetected isolates correspond exclusively to a certain geographic origin. Unfortunately, it was not possible to check if there was an impact of genotypes on the absence of detection, since the genotype of three of these accessions is still unknown.

Nine plants from three accessions (ITC0450, ITC1304, and ITC1586) known to be healthy tested negative. Accessions details are provided in Supplementary File S1. They were used as negative controls for this test. Thus, 100% of the diagnostic specificity was obtained.

**Figure 2:**
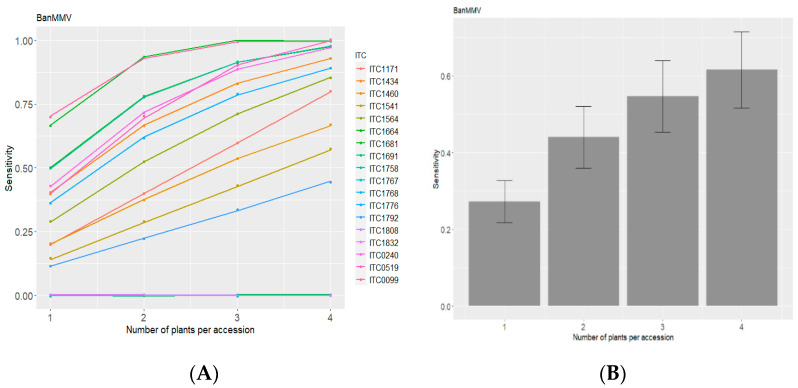
(**A**) Percentage of sensitivity of diagnostic of BanMMV from in vitro plants using 1, 2, 3, and 4 plants per combination for each accession. (**B**) Comparison of average sensitivity between combinations regardless of accessions. Error bar represent +/− standard deviation.

In addition, the results of up to four plants per accession were combined in order to follow the recommendations of the technical guidelines [24] for indexing from banana leaves, which recommend the testing of four individual plants per accession. The results are presented in Figure 2.

Figure 2 illustrates that the diagnostic sensibility increased when the number of plants per combination increased, considering the virus detected when at least one plant is positive for the combination. Overall, average diagnostic sensitivity per accession varied from 45 to 100% when taken into account all the combinations of four plants/accession (except two accessions that remained at 0% and which corresponded to false-negative results), considering a positive if at least one plant among the four tested positive. Figure 2B shows that the average diagnostic sensitivity varied from 27 to 61%, respectively, for combinations with one to four plants. The simulated diagnostic sensitivity increased with the number of plants tested in the combinations. The highest sensitivity, being 61%, was reached with combinations of four plants for each accession of the six accessions that would have at least one positive combination with four plants. It has been also shown that for two out of the 19 accessions (ITC1171 and ITC1691), 100% of the detection was achieved with combinations of three plants/accession.

#### 3.1.2. Comparison of Virus Detection after Thermotherapy from Corm Tissues of In Vitro Plants and from Leaves of Greenhouse Plants Using an IC-RT-PCR Assay

According to Table 2, BanMMV was detected from either corm tissues (in vitro tissues) or leaves of greenhouse plants, even after heat treatment, meristem culture, and a greenhouse acclimatization step. A success rate of 73% (per plant) for banana sanitation has been recorded in this study, confirming that thermotherapy in combination with meristem culture does not have a 100% efficacy for eradicating the virus.

Tests from tissue surrounding the meristem were carried out directly after one month of heat treatment for the meristem excision. The percentage of in vitro plantlets that tested negative was 61% (25 plantlets out of 41 tested). We must take into account also that the false-negative rate when testing such tissue from in vitro plant is quite important. On the other hand, leaf tests from fully developed plants were carried out after thermotherapy, meristem culture, and a greenhouse acclimatization of these plants for at least three months. Thirty of the 41 tested plantlets tested negative, showing a banana sanitation rate of 73%.

As a result, we noticed that 22 out of 41 tested plants (53.7%) presented similar test results between in vitro and greenhouse samplings, corresponding to 18 negative and four positive results, whereas discrepancies were observed for 19 out of 41 tested plants (46.3%). For seven plants, the corm testing was negative, and the greenhouse testing was positive, while the opposite results were observed for 12 plants.

### 3.2. Diagnostic Sensitivity of RT-PCR from Corms and Leaves of In Vitro Plants

#### 3.2.1. PCR Results of Infected and Healthy Banana Accessions

In vitro tissues from either the basal section or leaves were sampled. RT-PCR was carried out on the purified total RNA extracted from these samples. The testing was conducted on seven BanMMV-infected banana accessions (23 plants) and four healthy accessions known to be BanMMV-virus-free (16 plants). Results are detailed in Table 3.

Out of these 23 plants, fifteen tested positive for BanMMV presence when sampling in vitro tissues from either the base part or the leaves, reporting a diagnostic sensitivity of 65% from these two sections. The percentage of detection per accession varied from 0% to 100%. Among the seven infected accessions, three showed 100% of virus detection from both tissues. One accession (ITC0528) showed no virus detection from any sample, but the virus presence was based on the observation of a flexuous virus by electron microscopy. A lack of inclusivity of the tested primers could be the origin of the negative result and was further investigated (the healthy accessions all tested negative, reporting a diagnostic specificity of 100% through this molecular test).

**Figure 3:**
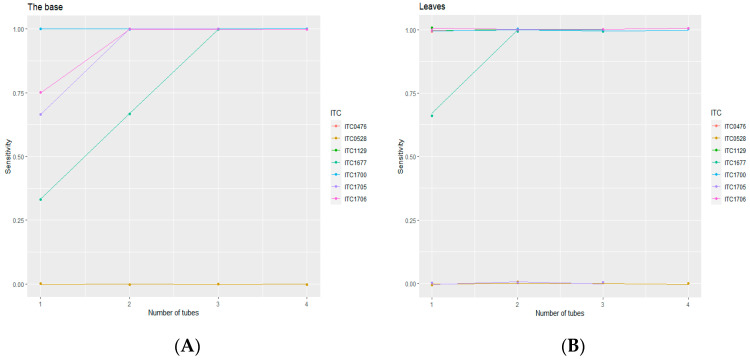
Diagnostic sensitivity of the test detecting BanMMV from two different in vitro tissues using combinations of plants per accession: (**A**) from the base (corm + meristem); (**B**) from leaves.

Then, the results on individual plants were combined with up to four plants per accession (Figure 3). The diagnostic sensitivity increased when the number of plants per combination increased, regardless of the sampled tissue. Interestingly, Figure 3B showed that a plateau was reached with combinations of two plants/accession, showing a sensitivity of 100% through the test conducted from leaf tissues. This means that, for the tested accessions, 100% diagnostic sensitivity is reached when considering individual RT-PCR tests of at least two plants per accession.

#### 3.2.2. Detection of BanMMV by High-Throughput Sequencing Test on In Vitro Plants

To determine whether HTS could consistently detect BanMMV from in vitro plants, we selected nine banana accessions previously tested by RT-PCR and corresponding to seven infected and two healthy ones. To mimic the indexing protocol relying on the analysis of four plants pooled for each accession [10] and to take into account its higher cost, HTS was only applied on mixes of four plants per accession from either the base or leaves. Results are detailed in Table 4.

To check for cross-sample contamination, 10,623,038 reads, 10,151,628 reads, and 9,080,280 reads were generated from the first external alien control (corresponding to wheat sample infected by BYDV), among which 49,563 reads, 41,868 reads, and 25,845 reads corresponded to BYDV. In addition, 10,563,714 reads, 7,808,992 reads, and 10,736,536 reads were generated from the second external alien control (corresponding to tomato samples infected by PepMV), among which 53,331 reads, 54,681 reads, and 73,675 reads corresponded to PepMV. A very low PepMV contamination was observed through this test, with a maximum contamination ratio of 18 on banana samples (for ITC1677) and 60 for the BYDV alien control (on a maximum of 73,675 reads), corresponding to a contamination ratio of 1:1227. These results underlined the interest of adding an alien control to monitor the cross-contamination between samples.

In addition, no BanMMV contamination was observed in the two negative controls nor in the alien controls. The risk of detecting cross-sample contamination was indeed low for BanMMV, since all BanMMV-infected samples presented a very low proportion of BanMMV sequences (a maximum of 5135 reads on nine million reads for sample ITC0476 and a minimum of 46 for ITC0528-Pl(3)). This also underlined the importance of using positive controls at a low concentration in routine detection by HTS tests. Supplementary data from the sequencing are presented in Supplementary File S6. BanMMV reads were detected in all the infected samples. The DSE and DSP of HTS on pooled plant tissues were both at 100% from either the base or the leaves.

In same line with the RT-PCR results, the average DSE from the base was the same as the one from the leaves of the same in vitro plant. This sensitivity was similar when comparing HTS to RT-PCR for the same samples.

A BanMMV infection was also detected by HTS on the accession ITC0528, although the accession tested negative by RT-PCR. There were four mismatches between the BanMMCP2 primer sequence and the sequence of the isolate, explaining the false-negative result obtained by RT-PCR (unpresented results).

#### 3.2.3. Identification of a New Betaflexiviridae Species Infecting *Musa* from ITC0528

A divergent Betaflexiviridae isolate with a reconstructed genome of 7364 bp was obtained by analysing the sequencing data from the corms and leaves of ITC0528, an accession belonging to *Musa* ornata Roxb. species. The pairwise alignment showed that the new genome sequence shared a pairwise identity of 62.7% at the nucleotide level with theBanMMV reference genome (GenBank accession NC_002729). Sequences from the new virus were not detected in the other accessions sequenced during this study nor in the more than 40 other accessions of Musa sp. sequenced at high throughput (Rong Wei, personal communication). Through the targeted RT-PCR and Sanger sequencing of amplicons, RdRp and CP sequences were confirmed. They presented 99.3% and 99.7% of identity, respectively, with the RdRp and CP consensus sequences generated by HTS.

The conserved domain assessment of the new species revealed the presence of all the plant viral domains of the Betaflexiviridae family (Table 5).

Two non-coding regions were found at the genome ends, 5′ UTR and 3′ UTR of 51 and 126 nt, respectively. The comparison of these values with members of the genus Foveavirus, for instance, belonging to the Betaflexiviridae family (5′UTR of 33–72 nt and 3′UTR of 176–312 nt) or with the BanMMV reference genome (GenBank accession NC_002729) (69 nt 5′ UTR and 77 nt 3′UTR and), showed that the genome reported lacks less than 50 nucleotides at its 3′ end and a maximum of 200 nt at its 5′ end [25].

Further annotation revealed that the new contig has the typical genome organization of Betaflexiviridae members (Table 6). The genome organization and ORF sizes are similar to BanMMV but with a protein identity of a maximum of 65% for RdRp.

At the nucleotide level, the highest percentages of identity between the sequence of the new species and a BanMMV sequence were 58% (with FJ179164.1) for complete CP (*n* = 14) and 64% (NC_002729) for complete RdRp (*n* = 3). At the protein level, the highest percentages of identity between the sequence of the new species and a BanMMV sequence were 57% for CP (with FJ179163.1, AY319333, and AY319332) and 65% for RdRp (NC_002729). Throughout the Betaflexiviridae family, isolates of different species should have less than about 72% nt identity (or 80% aa identity) between their respective CP and polymerase genes [25]. In addition, the new genome has the conserved domains and the five ORFs of Betaflexiviridae members.

**Figure 4:**
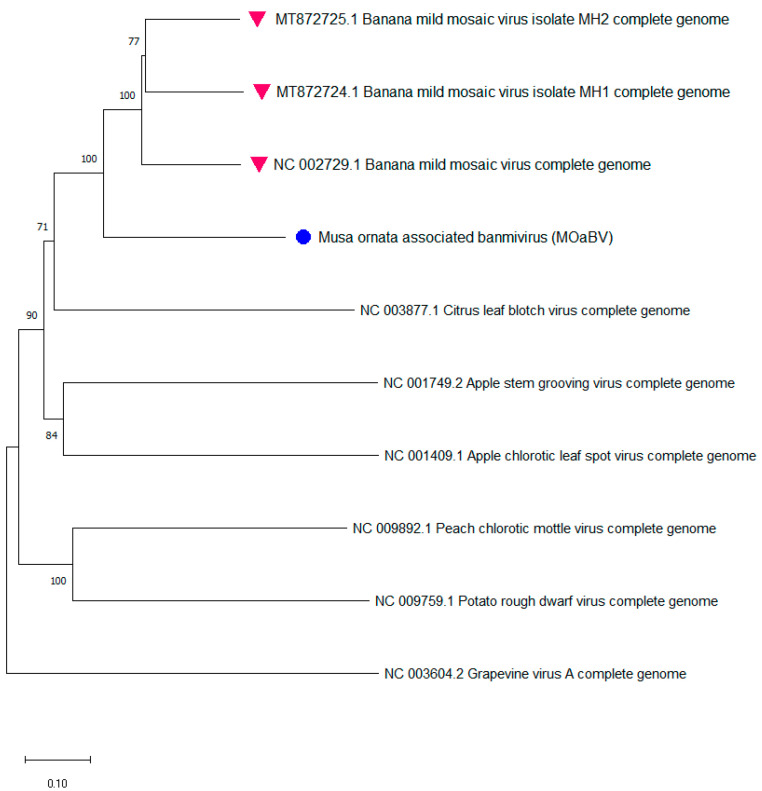
Neighbor-joining method phylogenetic tree inferred from full genomes of nt sequences of the new species from this study, complete genomes of BanMMV, and members from different genera of the Betaflexiviridae family. The purple triangle denotes the 3 existing complete genomes of BanMMV. The cyan dot denotes the new species identified throughout this work. Branches were bootstrapped with 1000 replications. The scale bar indicates the number of substitutions per site.

A phylogenetic tree including the sequenced genome sequence of the new isolate and the full genomes of some viruses from different genera of the Betaflexiviridae family (Figure 4) showed the clustering of the new species into a group with BanMMV, confirming its taxonomical position within this family of viruses and close to BanMMV, potentially belonging to the Banmivirus genus. Therefore, the sequenced isolate could be considered as a putative new species infecting *Musa ornata*, tentatively named *Musa ornata associated Banmivirus* (MoaBV).

This putative new species might be the second species of the proposed Banmivirus genus (with BanMMV currently as the unique species) within the Betaflexiviridae family.

## 4. Discussion

In this publication, several detection tests have been applied on *Musa* in vitro plants. The DSE of IC-RT-PCR and RT-PCR applied on individual plants were 38% and 65%, respectively. For the same virus, De Clerck et al. [10] observed only 20% diagnostic sensitivity using a one-step RT-PCR from crude extracts on a single leaf per plant. Our results improved the DSE but also underlined the challenge to reliably detect BanMMV from in vitro plants.

The false-negative results can arise for different reasons. First, the uneven distribution of viruses in plantlets. For instance, Helliot et al. [26] have reported the heterogeneous distribution of CMV and BSV viral particles in the meristematic cells of *Musa* plantlets. In a similar context, Spiegel et al. [27] have described the uneven distribution of the tobacco streak virus (TSV) in the shoot and root systems of infected strawberry plantlets and that up to 30% of the progeny plants tested negative by ELISA. Plotnikov et al. [28] have shown that there is a correlation between the viral load of the cucumber green mottle mosaic virus (CGMMV) and the part of the cucumber plant (r = 0.99). Moreover, the same authors have found that high values of CGMMV concentration in the cucumber leaves of greenhouse plants were observed on the middle (46%) and lower (36%) leaves. Jones et al. [29] also highlighted heterogeneity in virus composition and concentration in different tissues of the plant. This heterogeneity is taken into account for the BanMMV testing of plants in a greenhouse, as it is recommended to sample the three youngest leaves for each plant to carry out a complete indexing of an accession [24]. The better results obtained from the base of the plant compared to the corm can be explained by the heterogeneous distribution of the virus in the plants. Indeed, even if some plant RNA viruses are known to infect meristematic cells [30,31], many viruses do not invade or are at very low concentrations in the meristem and surrounding young tissue, causing false-negative results from corm sampling.

Another hypothesis that can explain this phenomenon is the fact that viral titers can be very low, e.g., under the limit of detection, in in vitro plant tissues. Similarly, Umber et al. [15] have outlined the low viral titers in yam in vitro plants, possibly below detection thresholds. Moreover, Azad et al. [32] have suggested that the absence of vascular elements in meristem cells in potatoes might be the reason for the low virus concentration in the meristem. This low concentration could also explain the observed results during post-therapy testing that are discussed hereunder.

An effect of the treatment applied on the in vitro plant was observed on the ability to detect BanMMV. Indeed, the detection by IC-RT-PCR of BanMMV from corm tissue surrounding the meristem was still more challenging when sampling it after thermotherapy. Although the same results between in vitro corm and greenhouse leaf testing were obtained for 54% of the plants, the DSE dropped to 36% (four in vitro plants detected positive on the 11 plants tested positive in the greenhouse). A very low and heterogenous concentration of BanMMV in these tissues could explain the observed results. In addition, corm tissue surrounding the meristem from 12 plants tested positive while the plant tested negative in the greenhouse. These 12 positive results might be explained by the fact that the virus was present in the plant tissue surrounding the meristem but absent in the meristem, which is the objective of the thermotherapy (meristem-free explants). Thermotherapy can reduce virus movement towards the meristem [31]. In fact, the exposure of infected plants to high temperature could eliminate synthesis of both coat protein and movement proteins. This would likely restrict the cell-to-cell movement of a pre-existing virus [33]. It also allows the reduction of the viral replication rate in vascularized tissues [15]. However, several studies have highlighted the limited efficiency of the use of thermotherapy alone [15,31]. In addition, Umber et al. [15] have reported that meristem excision favors the regeneration of healthy plantlets from virus-free totipotent cells. Furthermore, it has been described that testing the virus status after greenhouse acclimation remains important, as the virus may be suppressed and not completely eliminated in some treatments at the tissue culture stage [31]. The sampling of this tissue for BanMMV detection is therefore not recommended from an in vitro plant. The sampled tissue influenced the performance of BanMMV testing by RT-PCR from RNA extracts of in vitro plants. The amplification from the base (including the meristem) or the three youngest leaves of individual plants presented a similar DSE of 67%. Nevertheless, for one accession (ITC1705), no BanMMV detection was recorded from leaves, whereas 67% of DSE was obtained from the base. This might be explained by the low and/or heterogenous viral titer in leaf tissues of this accession that could possibly be below detection thresholds [15]. Thus, a slightly better sensitivity might probably be obtained from the base compared to the leaves for some accessions. Nevertheless, sampling from the base causes the death of the plant, as these tissues contain the meristem. Thus, it is not possible to regenerate the plant after the experiment. This can be problematic in the case of precious plants or can delay the testing until after in vitro multiplication.

Whatever the sampled tissue and the detection test applied, testing several plants individually for each accession and considering the accession as infected if a single plant is positive improved the DSE of the test. This decision reduced the false-positive rate to zero for the healthy accessions. This observation confirmed for in vitro plants the recommendations of the guidelines [24] for virus indexing on greenhouse *Musa* plants. These guidelines recommend the testing of four individual plants per accession, as the distribution of BanMMV can be heterogeneous between the plants. Therefore, any BanMMV testing from in vitro plants should also include at least four plants, even if for some accessions 100% DSE was obtained by RT-PCR considering only two in vitro plants.

The proportion of false negative per individual plant was also variable between accessions. Although a DSE of 100% was reached when testing the base of four individual plants, these results should be further confirmed on a larger number of accessions.

The HTS test was applied on a pool of four plants per accession and the sampling of either three leaves per plant or the complete base to address the virus heterogeneity in and between the plants. Pooling samples also lowered the cost of the HTS technologies, which is still much higher than RT-PCR. In addition, the very high analytical sensitivity of HTS represented an asset for detecting viruses present in very low concentrations [34,35].

HTS technologies showed excellent performances from pools of four plants, achieving 100% of DES from the base or the leaves of the accessions, although with a very low proportion of BanMMV sequencing reads for some accessions. The DSP of HTS was 100%, as it was for the PCR-based technique. In addition, the very high inclusivity of HTS was again demonstrated with the ability to identify a putative new species of Betaflexiviridae and an isolate of BanMMV presenting mismatches in the primer sequence. Ultimately, thanks to their untargeted nature, HTS technologies could also be used in the future to detect other viruses infecting the in vitro plants of a *Musa* accession. In addition, the use of an alien control would strengthen the reliability of the results by monitoring the contamination burden.

## 5. Conclusions

In summary, for detecting BanMMV from in vitro tissues, two tests can be recommended. First, HTS technologies could be applied on the RNA extracted from pooled leaves or bases from at least four plants per accession. Alternatively, if HTS technologies are not available or too expensive, RT-PCR could be applied on the total RNAs extracted from the base or, if the plant cannot be destroyed, from three leaves of at least four individual in vitro plants (four biological replicates) per accession. These encouraging preliminary results warrant further application and evaluation of BanMMV detection on a larger panel of accessions and the extension of the proposed methodology to other viruses infecting *Musa* plants for which greenhouse cultivation is also mandatory.

## Figures and Tables

**Table 1:** BanMMV detection by IC-RT-PCR from corm tissue of infected in vitro plants (19 accessions).

Accession Code	Number of Tested Plants	Plants Tested Positive	Plants Tested Negative	Diagnostic Sensitivity
ITC0099	10	7	3	70%
ITC0240	7	3	4	≈43%
ITC0519	5	2	3	40%
ITC1171	5	1	4	20%
ITC1434	10	4	6	40%
ITC1460	10	2	8	20%
ITC1541	7	1	6	≈14%
ITC1564	7	2	5	≈29%
ITC1664	10	8	2	80%
ITC1681	6	5	1	≈83%
ITC1691	5	0	5	0%
ITC1758	10	10	0	100%
ITC1767	5	0	5	0%
ITC1768	5	0	5	0%
ITC1776	11	5	6	≈45%
ITC1792	9	2	7	≈22%
ITC1808	5	0	5	0%
ITC1832	5	0	5	0%
ITC1833	5	0	5	0%
**Total**	**137**	**52**	**85**	**38%**

The healthy accessions used as negative controls during this experiment were: ITC0450, ITC1304, and ITC1586, and negative results were always obtained.

**Table 2:** Comparison of BanMMV detection between in vitro corm tissues and greenhouse leaf samples of the same 41 banana plants (21 accessions) using IC-RT-PCR. “+” refers to a positive result; “−“ refers to a negative result; the healthy accessions used as negative controls through this experiment were ITC0245, ITC0654, and ITC1120, and they tested negative.

Accession Code	Tested Plant	Corm Results	Leaf Results (in Greenhouse)
ITC0099	1	−	−
ITC0240	1	−	−
ITC0321	1	−	−
ITC0519	1	−	−
	2	−	−
ITC1380	1	+	−
	2	+	−
	3	+	−
ITC1434	1	+	−
	2	−	−
ITC1460	1	+	−
	2	−	−
ITC1564	1	−	−
	2	+	−
	3	+	−
ITC1664	1	+	+
	2	+	−
	3	+	+
ITC1681	1	+	+
	2	−	+
	3	−	+
ITC1734	1	−	−
	2	−	−
ITC1748	1	−	−
	2	−	−
	3	−	+
ITC1752	1	−	+
ITC1758	1	−	+
ITC1767	1	−	−
ITC1768	1	+	−
	2	−	−
	3	−	−
ITC1776	1	−	+
ITC1792	1	+	−
	2	+	−
	3	+	−
ITC1808	1	+	+
	2	−	−
ITC1831	1	−	+
	2	−	−
ITC1857	1	−	−

**Table 3:** Testing of BanMMV infection from either basal section or leaves of infected in vitro plants. DSE = diagnostic sensitivity. Tested + and – mean BanMMV detected or not detected respectively.

Accession Code	Nb. of Tested Plants	Status	The Base of the Plant			Leaves of the Same Plant		
			Plants Tested +	Plants Tested −	DSE	Plants Tested +	Plants Tested −	DSE
ITC0476	2	Infected	2	0	100%	2	0	100%
ITC0528	4	Infected *	0	4	0%	0	4	0%
ITC1129	3	Infected	3	0	100%	3	0	100%
ITC1677	3	Infected	1	2	33%	2	1	67%
ITC1700	4	Infected	4	0	100%	4	0	100%
ITC1705	3	Infected	2	1	67%	0	3	0%
ITC1706	4	Infected	3	1	75%	4	0	100%
ITC0245	4	Healthy	0	4	-	0	4	-
ITC0654	4	Healthy	0	4	-	0	4	-
ITC1120	4	Healthy	0	4	-	0	4	-
ITC1586	4	Healthy	0	4	-	0	4	-

*: The infection status of this accession was determined by electron microscopy and the observation of filamentous particles.

**Table 4:** HTS results of BanMMV detection from RNA extracts of the base or the leaves of in vitro plants. The number of reads per sample ranged between 6,499,298 and 10,357,840. + and − mean BanMMV detected or not detected respectively.

Accession	Tissue	Individual	RT−PCR Result	Number of Reads Mapped to BanMMV Sequences (with 20% of Mismatches)	1st Alien Control (BYDV)	2nd Alien Control (PepMV)	Total Number of Reads
ITC0476	Base	Mix	+	5135	0	2	9,007,880
	Leaves	Mix	+	1822	0	3	6,499,298
ITC0528	Base	Pl(1) Pl(2) Pl(3) Mix	− − − −	49 67 46 311	0 1 0 0	2 0 0 0	9,211,966 10,346,472 8,606,200 10,357,840
	Leaves	Pl(1) Pl(2) Pl(3) Mix	− − − −	162 193 171 715	0 0 0 0	2 1 1 3	9,419,300 8,544,882 7,473,540 9,139,050
ITC1129	Base	Mix	+	667	0	2	9,791,204
	Leaves	Mix	+	683	0	3	8,717,902
ITC1677	Base	Mix	+	282	0	2	9,671,458
	Leaves	Mix	+	109	1	18	8,755,638
ITC1700	Base	Mix	+	2260	0	1	9,777,910
	Leaves	Mix	+	3499	0	0	8,245,604
ITC1705	Base	Mix	+	477	0	0	8,759,412
	Leaves	Mix	+	384	1	4	8,711,232
ITC1706	Base	Mix	+	1620	0	5	9,822,322
	Leaves	Mix	+	625	0	15	6,335,434
ITC1586 *	Base	Mix	−	0	0	0	8,414,752
ITC0654 *	Leaves	Mix	−	0	0	0	8,443,436
BYDV−infected alien	Leaves	Pl(1) Pl(2) Pl(3)	− − −	0 0 0	49,563 41,868 25,845	2 60 2	10,623,038 10,151,628 9,080,280
PepMV−infected alien	Leaves	Pl(1) Pl(2) Pl(3)	− − −	0 0 0	2 0 0	53,331 54,681 73,675	10,563,714 7,808,992 10,736,536

* ITC1586 and ITC0654 are healthy accessions used as negative controls; Pl refers to plant; Mix corresponds to a mix of four RNA extracts, each coming from one plant of the accession.

**Table 5:** List of conserved domains of the new species predicted by NCBI Conserved Domain Search.

Name	Accession	Description	Interval	E-value
Vmethyltransf	pfam01660	Viral methyltransferase	181–1125	2.18 × 10^−65^
RdRP_2 super family	cl03049	RNA dependent RNA polymerase	4096–5229	1.86 × 10 ^−37^
Viral_helicase1 super family	cl26263	Viral (Superfamily 1) RNA helicase	2911–3630	3.54 × 10 ^−09^
Peptidase_C23 super family	cl05111	Carlavirus endopeptidase	2398–2655	1.28 × 10 ^−03^
Viral_helicase1	pfam01443	Viral (Superfamily 1) RNA helicase	5372–5956	7.06 × 10 ^−36^
Plant_vir_prot	pfam01307	Plant viral movement protein	5945–6238	2.25 × 10 ^−23^
Flexi_CP super family	cl02836	Viral coat protein	6690–7094	6.96 × 10 ^−41^
7kD_coat	pfam02495	7kD viral coat protein	6216–6380	1.26 × 10 ^−04^

**Table 6:** BLASTP results of the five ORFs of the new Betaflexiviridae species.

	Interval	Maximum Protein Identity (Protein-Protein BLAST)	Organism	Accession
ORF1	52 -> 5268	64.6%	RNA-dependant RNA polymerase (Banana mild mosaic virus)	QVD99720.1
ORF2	5261 -> 5935	46%	Triple gene block protein 2 (Banana mild mosaic virus)	QVD99726.1
ORF3	5936 -> 6274	58%	Triple gene block protein 3 (Banana mild mosaic virus)	QVD99727.1
ORF4	6192 -> 6398	64%	Triple gene block protein 4 (Banana mild mosaic virus)	QVD99723.1
ORF5	6471 -> 7238	60.8%	Coat protein (Banana mild mosaic virus)	ACN91624.1

## Data Availability

The nucleotide sequence of the new species was successfully deposited in GenBank under the following Genbank accession number, OK721096, and the partial sequences obtained by RT-PCR and Sanger sequencing were deposited under the following Genbank accession numbers OL412396 and OL412397, respectively, for RdRp and CP sequences. All raw reads produced and used in this study were submitted to the NCBI’s Sequence Read Archive (SRA) under Bioproject PRJNA784940.

## Supplementary Materials

The following are available online at https://www.mdpi.com/article/10.3390/plants11020226/s1, Supplementary File S1: accessions list, Supplementary File S2: BanMMV detection protocol, Supplementary File S3: sequences list, Supplementary File S4: primers and PCR programs used for Sanger sequencing, Supplementary File S5: photo gel, Supplementary File S6: HTS supplementary.
