# Supplementary material for: Detection of Banana Mild Mosaic Virus in Musa In Vitro Plants: High-Throughput Sequencing Presents Higher Diagnostic Sensitivity Than (IC)-RT-PCR and Identifies a New Betaflexiviridae Species
Source: Plants (Basel). 2022 Jan 15;11(2):226. doi: 10.3390/plants11020226 (PMC8777661; doi:10.3390/plants11020226)
Supplement: Supplementary file 1 [file plants-11-00226-s001.zip › Supplementary File S4- Primers and PCR programs used for sanger sequencing.pdf]

Table. Primers and corresponding PCR programs used for the sanger sequencing in this study. The primers pairs in bold have been used to amplify the RdRp of the new species. The remaining two pairs have been used to amplify the CP of the species.

Primer name	Sequence (5'-3')	PCR program
**73F1**	TCCATTCTCGCTGCAATTAG	94°C for 30s→(94°C for 15s, 50°C for 30s, 72°C for 2min30s) X 40 cycles→ 72°C 10min
**510R1**	TCGGGCTGAAAACAGGTTGA	
**318F2**	TGACACAGCTAGGGAGTTACTTG	94°C for 30s→(94°C for 15s, 52°C for 30s, 72°C for 2min30s) X 40 cycles→ 72°C for 10min
**1026R2**	TGAATGTGAAAATATGGTGTAGGGT	
**940F3**	GGTGACATTTTGAGGACACGC	
**1949R3**	TCAGTATCCACAATTTCTACAAACTCA	
**1726F4**	AGCGAGAATTCTGACACTGCA	
**2739R4**	TGGCCCAATACTTCCAATTTCA	
**2625F5**	TCAAGAAGGTCATGGGTTGGA	
**3346R5**	TGTCACTGTCGTAGGAAGCT	
**3250F6**	CCCCCAGGCTATCTTGATTTGA	
**4343R6**	GCCATTTCTTCACCCATGCG	
**4125F7**	TGGACACATCACATCAATGCA	
**5489R7**	ATCCAGCCACTCCATGCAC	
6557F8	TGTGAATAGAACTTGTGTGTGTGA	94°C for 30s→(94°C for 15s, 48°C for 30s, 72°C for 2min30s) X 40 cycles→ 72°C for 10min
6941R8	GGCGCTTGAACCATTGTGAT	
6827F9	AGAACTTGATTGGATTGGGGACT	94°C for 30s→(94°C for 15s, 52°C for 30s, 72°C for 2min30s) X 40 cycles→ 72°C for 10min
7406R9	TCAACGAACATTTTTACGCGT	
