# Supplementary material for: Detection of Banana Mild Mosaic Virus in Musa In Vitro Plants: High-Throughput Sequencing Presents Higher Diagnostic Sensitivity Than (IC)-RT-PCR and Identifies a New Betaflexiviridae Species
Source: Plants (Basel). 2022 Jan 15;11(2):226. doi: 10.3390/plants11020226 (PMC8777661; doi:10.3390/plants11020226)
Supplement: Supplementary file 1 [file plants-11-00226-s001.zip › Supplementary File S5- Photo gel.pdf]

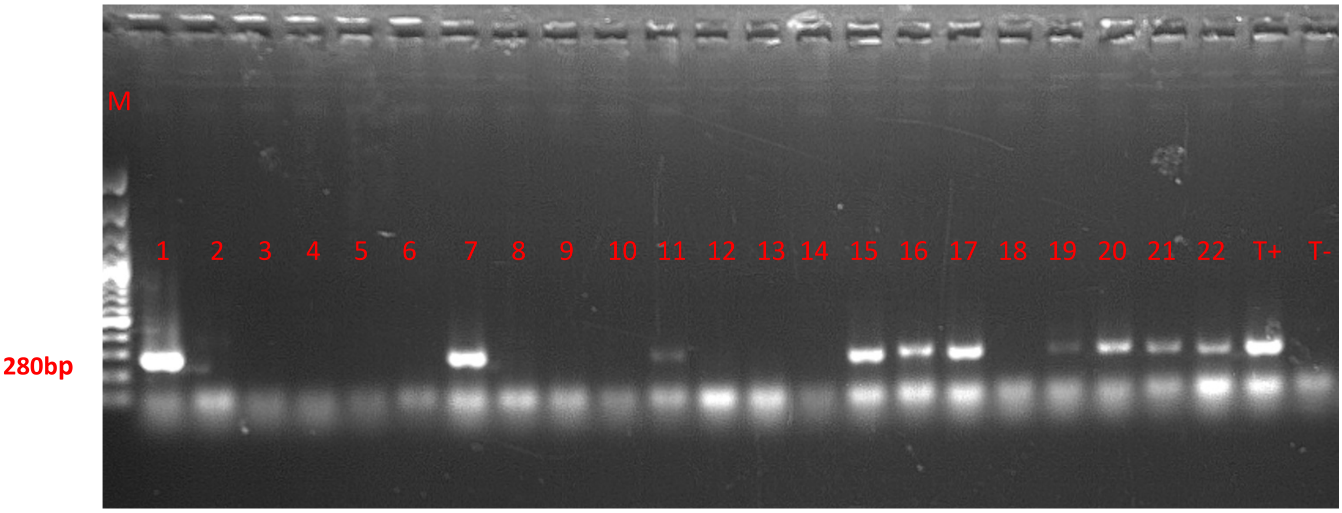

With M : 100bp molecular weight marker, T+ refers to positive control and T- refers to negative control (healthy banana).

Samples; from 1→7: ITC1564; from 8→14: ITC1541; from 15→22: 8 plants from ITC 0099
